# Effects of Dietary Sterol-Enriched Yeast Hydrolysate on Ovarian Development, Reproductive Endocrine Status, Immune-Inflammatory Responses, Antioxidant Indices, and Egg Quality in Late-Laying Yellow-Feathered Broiler Breeder Hens

**DOI:** 10.3390/ani16172750

**Published:** 2026-09-02

**Authors:** Haoran Si, Zhifang Shi, Mengyun Li, Yining Zhang, Bo Fan, Xuanyang Li, Yongzhen Li, Xiaotong Wang, Jian Liu, Lei Xi

**Affiliations:** 1 Animal Science, Xinjiang Agricultural University, No. 311 Nongda East Road, Urumqi 830052, China; 19837309151@163.com (H.S.); 13663800339@163.com (Y.Z.); fbbb419@163.com (B.F.); 2 Animal Science, Henan University of Animal Husbandry and Economy, No. 6 Longzihu North Road, Zhengzhou 450046, China; shizhifang83158@163.com (Z.S.); limengyun1@163.com (M.L.); 201006@hnuahe.edu.cn (X.L.); yongzhenli@hnuahe.edu.cn (Y.L.); xtwang2026@163.com (X.W.); 13513808848@163.com (J.L.); 3 Henan Provincial Engineering Research Center for Healthy Livestock and Poultry Farming and Intelligent Equipment, No. 6 Longzihu North Road, Zhengzhou 450046, China

**Keywords:** sterol-enriched yeast hydrolysate, broiler breeder hen, ovarian development, reproductive endocrinology, antioxidant status

## Abstract

This study evaluated graded dietary supplementation with sterol-enriched yeast hydrolysate (SYH) in late-laying yellow-feathered broiler breeder hens. A total of 720 55-week-old hens received 0, 0.2, 0.5, or 0.8 g/kg of SYH for six weeks after a one-week adaptation period. SYH is a composite product containing sterols, mannan, nucleotides, and amino nitrogen; therefore, the observed effects cannot be attributed to sterols alone. Eggshell strength increased at 0.5 and 0.8 g/kg, whereas fertility and hatchability were unaffected. The 0.5 g/kg group showed the highest ovarian index values and the greatest quantities of 5–12 mm yellow follicles. The 0.8 g/kg group showed the highest serum reproductive hormone and immunoglobulin concentrations, together with the highest reactive oxygen species and cytokine concentrations. Hepatic and ovarian superoxide dismutase activity increased selectively, whereas most other antioxidant indices were unchanged. Ovarian *FSHR*, *CYP19A1*, and *VDR* mRNA expression showed concentration-specific patterns. The strongest peripheral endocrine response at 0.8 g/kg did not coincide with the most favorable ovarian developmental response. Under the conditions of this study, 0.5 g/kg represents a candidate inclusion concentration for further validation rather than a confirmed optimum.

## Simple Summary

Late-laying broiler breeder hens often show declining reproductive performance. This study evaluated dietary sterol-enriched yeast hydrolysate (SYH; 0, 0.2, 0.5, or 0.8 g/kg) in 720 hens over a six-week feeding period. The 0.5 g/kg treatment improved eggshell strength and was associated with the highest ovarian index and the greatest quantities of 5–12 mm yellow follicles. In contrast, 0.8 g/kg elicited the strongest endocrine and immune responses but also the highest increases in oxidative and inflammatory markers. Hepatic and ovarian SOD activity increased selectively, whereas fertility and hatchability were unaffected. Under the conditions of this study, 0.5 g/kg provided the most balanced response and should be considered a candidate inclusion concentration for further validation.

## 1. Introduction

In intensive production systems, broiler breeder hens in the late laying period commonly exhibit declining ovarian function, reduced follicular reserves, variable eggshell quality, and accumulated oxidative stress, which may compromise hatching-egg utilization and offspring quality. Follicular recruitment and ovulation are regulated by the hypothalamic–pituitary–gonadal axis. Hormones such as follicle-stimulating hormone (FSH), luteinizing hormone (LH), estradiol (E2), and progesterone (P4), together with ovarian genes including *FSHR* and *CYP19A1*, jointly regulate follicular growth, selection, and steroidogenesis [1,2,3]. Redox status is also closely linked to immune homeostasis; excessive reactive oxygen species can damage ovarian cells, whereas appropriate immune and antioxidant regulation may help maintain physiological function during the late laying period [4,5].

Yeast cultures and their derived fractions contain bioactive constituents such as peptides, oligosaccharides, nucleotides, vitamins, and sterols. Previous studies have shown that yeast culture can improve eggshell strength, albumen height, Haugh unit, and hatchability in aged breeder layers while enhancing total antioxidant capacity and immune-related indices [6,7]. Comi et al. reported that dietary Saccharomyces cerevisiae hydrolysate improved growth performance during the finishing phase in broilers and downregulated pro-inflammatory cytokine gene expression, including tumor necrosis factor-α (TNF-α), interleukin-1β (IL-1β), and interleukin-6 (IL-6) [8]. Park et al. found that brewer’s yeast hydrolysate improved laying performance, egg quality, nutrient digestibility, and excreta microflora in laying hens [9]. Existing research has focused primarily on yeast cultures, yeast fermentation products, yeast cell wall polysaccharides, and conventional yeast hydrolysates. Because these products differ substantially in terms of cell wall components, soluble metabolites, and sterol content, their biological effects cannot be considered interchangeable. Systematic information remains limited on the concentration-response effects of a yeast hydrolysate with a defined total sterol content in late-laying yellow-feathered broiler breeder hens and on whether changes in peripheral reproductive endocrine status are concordant with local ovarian function and follicular reserves.

We hypothesized that sterol-enriched yeast hydrolysate (SYH) would influence follicular development and egg quality in late-laying broiler breeder hens by modulating immune and redox homeostasis and the hypothalamic–pituitary–gonadal axis, and that the biological response would vary with the supplementation concentration because the product contains multiple bioactive components. Accordingly, this study evaluated the effects of graded SYH supplementation on reproductive performance, immune indices, tissue antioxidant status, and the expression of ovarian-function-related genes.

## 2. Materials and Methods

### 2.1. Additive and Animal Management

The treatment additive was supplied by Hebei Ronghe Yeast Co., Ltd., and analyzed by an independent third-party laboratory (report no. NZZ20231001695-2; sample no. NZZ20231001695; submitted sample name: yeast sterol). The product contained 9.24% mannan, 3100 mg/100 g of total sterols, 2.0 g/100 g of amino nitrogen, and 3.64 g/kg of nucleotides. Based on a total sterol concentration of 31 mg/g, supplementation with 0.2, 0.5, and 0.8 g/kg of SYH corresponded to theoretical total sterol additions of 6.2, 15.5, and 24.8 mg/kg of diet, respectively.

The experiment was conducted at a commercial broiler breeder farm in Henan Province, China. Birds were managed according to the routine procedures of the farm. Hens received a restricted daily feed allowance of 120 g/hen, provided in four meals at approximately 05:00, 09:00, 13:00, and 15:00, while water was available ad libitum throughout the experiment. Environmental conditions were maintained as consistently as possible among treatments, including temperature (22–26 °C), relative humidity (50–70%), and photoperiod (15–16 h/day). The house was naturally ventilated, and hens were vaccinated according to the standard farm immunization program. Artificial insemination was performed weekly using semen supplied by the farm. The feeding trial lasted six weeks, from 55 to 61 weeks of age.

### 2.2. Experimental Design

The study was approved by the Animal Ethics Committee of Henan University of Animal Husbandry and Economy (approval no. HNUAHE ER2607D601). A total of 720 healthy 55-week-old yellow-feathered broiler breeder hens (SASSO SA51A strain; mean body weight, 2135.00 g; mean laying rate, 74.31%) were randomly assigned to four dietary treatments, with six replicate pens per treatment and 30 hens per replicate. The treatments consisted of a basal diet without SYH (control) or the basal diet supplemented with 0.2, 0.5, or 0.8 g/kg of SYH. SYH was added to and thoroughly mixed with the feed without replacing other dietary ingredients. Because the highest inclusion concentration was only 0.08%, its contribution to dietary energy and protein was considered negligible; therefore, no isoenergetic or isonitrogenous adjustment was made. The feeding period lasted six weeks after a one-week adaptation period. The basal diet was formulated according to the farm’s nutrient specifications (Table 1). Crude protein, crude fat, and calcium were determined according to GB/T 6432-2018 [10], GB/T 6433-2006 [11], and GB/T 6436-2018 [12], respectively. Metabolizable energy and available phosphorus were calculated using the Chinese Feed Composition and Nutritional Value Tables (32nd edition, 2021).

### 2.3. Sample Collection and Measurements

#### 2.3.1. Egg Quality Assessment and Collection of Hatching Eggs

On day 42, 15 hatching eggs were collected from each replicate (90 eggs per treatment), individually labeled, and incubated for 21 days in a fully automatic incubator (Yunfeng DF-508, Beijing, China). Incubation conditions were 37.8 °C and 55–60% relative humidity on days 1–19 and 37.2 °C and 70–75% relative humidity on days 20–21. Eggs were turned every 2 h through an angle of ±45° until transfer on day 19, after which turning was stopped. Eggs were candled on day 7 to identify fertile eggs, and infertile eggs were removed. Fertility, hatchability of eggs set, and hatchability of fertile eggs were subsequently calculated. Fertility and hatchability were calculated separately for each replicate pen, with the replicate pen serving as the experimental unit (*n* = 6 per treatment); individual eggs were treated as subsamples. All eggs were incubated in a single run under identical conditions.

Fertility (%) = number of fertile eggs identified by candling/number of eggs set × 100%.(1)

Hatchability of fertile eggs (%) = number of chicks hatched/number of fertile eggs × 100%.(2)

Hatchability of eggs set (%) = number of chicks hatched/number of eggs set × 100%.(3)

On day 42, four additional hatching eggs were selected from each replicate (24 eggs per treatment) for routine egg quality assessment. Measurements included egg weight, transverse and longitudinal diameters, eggshell strength, eggshell thickness, yolk weight, yolk color, and albumen height; egg shape index and Haugh unit were calculated. Egg diameters were measured using an electronic digital caliper. Egg weight, albumen height, yolk color, and Haugh unit were determined using a multifunctional egg quality analyzer (EMT-7300, Robotmation Co., Ltd., Tokyo, Japan). Eggshell strength was measured using an eggshell strength tester (YN-100, Hengtong Instrument Equipment Co., Ltd., Chaoyang, China), and eggshell thickness was measured using a 0–25 mm wall-thickness micrometer (Guilin Measuring & Cutting Tool Co., Ltd., Guilin, China). Yolk weight was determined using an electronic balance.

Egg shape index = transverse diameter/longitudinal diameter.(4)

#### 2.3.2. Serum Hormone and Immune-Related Indices

On day 42, three healthy hens with bodyweights close to the replicate mean were randomly selected from each replicate after a 12 h fast with free access to water (18 hens per treatment; 72 hens in total). Blood was collected once between 09:00 and 11:00. Approximately 10 mL of blood was collected from the wing vein of each selected hen as part of the terminal sampling procedure, allowed to clot for 30 min at room temperature, and centrifuged at 3500 r/min for 10 min at 4 °C. Serum was separated, snap-frozen in liquid nitrogen, and stored at −80 °C until analysis. Chicken-specific quantitative enzyme-linked immunosorbent assay (ELISA) kits (96T format; Jiangsu Meimian Industrial Co., Ltd., Yancheng, China) were used according to the manufacturer’s instructions to determine serum follicle-stimulating hormone (FSH; MM-0518O1), luteinizing hormone (LH; MM-1623O1), estradiol (E2; MM-0787O1), progesterone (P4; MM-60214O1), leptin (LEP; MM-37115O1), insulin-like growth factor-1 (IGF-1; MM-0510O1), immunoglobulin A (IgA; MM-0913O1), immunoglobulin M (IgM; MM-0912O1), immunoglobulin G (IgG; MM-0505O1), reactive oxygen species (ROS; MM-60120O1), interleukin-1β (IL-1β; MM-36910O1), interleukin-2 (IL-2; MM-0528O1), interleukin-6 (IL-6; MM-0521O1), tumor necrosis factor-α (TNF-α; MM-0938O1), and interferon-γ (IFN-γ; MM-0520O1) concentrations. All assays, including E2, P4, and ROS, were chicken-specific, and ROS were quantified by ELISA. FSH concentrations were reported in mIU/mL, consistent with the assay report. Following blood collection, the same hens were euthanized for tissue collection as described in Section 2.3.3.

#### 2.3.3. Ovarian Function and Gene Expression

Following blood collection, the same three hens from each replicate were weighed and euthanized by jugular exsanguination. The ovarian ligament was dissected to expose the ovary, and follicular development and morphology were examined. The ovary and oviduct were weighed to calculate the ovarian and oviduct indices. Ovarian follicles were classified as large, medium, or small yellow follicles, and follicles in each category were counted. Samples designated for molecular analysis were immediately frozen in liquid nitrogen and stored at −80 °C. Based on the diameter categories used during sampling, 5–12 mm yellow follicles were subdivided into 5–7, 7–9, and 9–12 mm groups. Previous avian studies have commonly classified yellow follicles of approximately 5–10 mm or 5–12 mm as prehierarchical small yellow follicles (SYF), whereas some studies have defined 9–12 mm follicles as large yellow follicles (LYF). Accordingly, the 5–7, 7–9, and 9–12 mm categories used here are operational subdivisions based on follicular diameter rather than a universally accepted avian hierarchical follicle classification. Because preovulatory hierarchical follicles > 12 mm (F1–F5) were not recorded, the present data primarily reflect changes in 5–12 mm yellow follicles during the recruitment and selection stage.

Ovary index (%) = ovary weight (g)/live body weight (g) × 100.(5)

Oviduct index (%) = oviduct weight (g)/live body weight (g) × 100.(6)

For RT-qPCR analysis, prehierarchical small yellow follicles (SYF; 6–8 mm in diameter) were specifically selected. Relative mRNA expression of *FSHR* and *VDR* was quantified in the granulosa compartment of ovarian follicles, whereas *CYP19A1* mRNA expression was quantified in the theca compartment, with β-actin used as the reference gene. The corresponding follicular compartments were collected separately for RNA extraction. Total RNA was extracted from the corresponding follicular samples using TRNzol Universal reagent (DP424, Tiangen Biotech Co., Ltd., Beijing, China). RNA concentration and purity were measured using a NanoDrop 2000 spectrophotometer (Thermo Fisher Scientific), and RNA integrity was evaluated by 1.5% agarose gel electrophoresis. Qualified RNA was reverse-transcribed into cDNA using the RevertAid First Strand cDNA Synthesis Kit (K1622, Thermo Fisher Scientific, Waltham, MA, USA). The reverse-transcription system, PCR reaction mixture, and thermal cycling program are provided in Supplementary Table S1. PCR was performed using an ABI 7500 Real-Time PCR System, and Ct values were obtained using ABI 7500 software. ΔCt was calculated as the Ct of the target gene minus that of the reference gene; ΔΔCt was calculated as the ΔCt of each treatment sample minus the mean ΔCt of the control group. Relative gene expression was calculated using the 2^−ΔΔCt method. Gene-specific primer sequences and reference accession numbers are listed in Table 2.

#### 2.3.4. Antioxidant Indices

After the abdominal cavity was opened, the liver, spleen, oviduct, and ovary were rapidly excised. Adherent fascia and fat were removed before the organs were weighed and organ indices were calculated.

Organ index (%) = organ weight (g)/body weight (g) × 100.(7)

Portions of the ovary, magnum region of the oviduct, and liver were rinsed with sterile phosphate-buffered saline (PBS), blotted dry with filter paper, frozen in liquid nitrogen, and stored at −80 °C. For tissue preparation, 0.1 g of ovarian tissue was homogenized on ice with 0.9 mL physiological saline to prepare a 10% tissue homogenate. Antioxidant indices measured in serum, liver, and ovary included glutathione peroxidase (GPx), malondialdehyde (MDA), superoxide dismutase (SOD), total antioxidant capacity (T-AOC), and catalase (CAT).

### 2.4. Statistical Analysis

Data were analyzed using IBM SPSS Statistics version 27.0 (IBM Corp., Armonk, NY, USA). The experiment followed a completely randomized design with four dietary treatments and six replicate pens per treatment, with the replicate pen considered the experimental unit. For variables measured in three hens within each replicate, individual values were averaged to obtain one replicate-level value before statistical analysis. Treatment effects were evaluated by one-way analysis of variance (ANOVA). When the homogeneity-of-variance assumption was satisfied and the overall treatment effect was significant, Duncan’s multiple range test was used for post hoc comparisons. When the homogeneity-of-variance assumption was violated, Welch’s ANOVA was used; a significant Welch test was followed by Games–Howell pairwise comparisons. Statistical significance was declared at *p* < 0.05.

## 3. Results

### 3.1. Egg Quality

Dietary SYH did not significantly affect egg weight, mean eggshell thickness, albumen height, Haugh unit, yolk weight, or egg shape index (*p* > 0.05; Table 3). Eggshell strength was highest in the 0.8 g/kg group and was significantly greater than in the control and 0.2 g/kg groups. The 0.5 g/kg group also had greater eggshell strength than the control but did not differ significantly from the 0.2 or 0.8 g/kg groups (*p* < 0.05). Yolk color was more intense in the control than in the 0.2 and 0.5 g/kg groups, whereas the 0.8 g/kg group had a higher yolk color score than the 0.5 g/kg group (*p* < 0.05).

### 3.2. Fertility and Hatchability

Dietary SYH did not significantly affect fertility or hatchability of eggs set (*p* > 0.05; Table 4). Hatchability of fertile eggs did not satisfy the homogeneity-of-variance assumption and was therefore evaluated using Welch’s ANOVA; no significant treatment effect was detected (*p* = 0.534). Fertility, hatchability of fertile eggs, and hatchability of eggs set were numerically higher in all SYH-supplemented groups than in the control, but none of these differences were statistically significant.

### 3.3. Serum Reproductive Hormones

Dietary SYH significantly affected serum P4, E2, FSH, LH, LEP, and IGF-1 concentrations (*p* < 0.05; Table 5). The 0.8 g/kg group showed the highest values for all six variables. Values in the 0.2 g/kg group were generally higher than those in the control and 0.5 g/kg groups, although this pattern was not consistent across all variables, indicating concentration-specific responses. Relative to the control, P4, E2, FSH, LH, LEP, and IGF-1 concentrations in the 0.8 g/kg group increased by 66.2%, 78.9%, 74.2%, 61.2%, 76.9%, and 89.4%, respectively.

### 3.4. Serum Immune- and Inflammation-Related Indices

Serum IgA, IgM, and IgG concentrations were significantly higher in the 0.8 g/kg group than in all other groups (*p* < 0.05; Table 6), representing increases of 48.9%, 82.7%, and 67.2%, respectively, relative to the control. ROS, IL-1β, IL-2, IL-6, TNF-α, and IFN-γ also reached their highest concentrations in the 0.8 g/kg group. For most indices, values at 0.2 g/kg exceeded those at 0.5 g/kg, whereas values at 0.5 g/kg were higher than or close to the control. Thus, the highest immunoglobulin concentrations at 0.8 g/kg occurred together with the highest ROS and cytokine concentrations.

### 3.5. Organ Indices and Ovarian Follicle Development

Dietary SYH did not significantly affect the liver or spleen indices (*p* > 0.05; Table 7). The ovarian index was significantly higher in the 0.5 g/kg group than in the control and 0.2 g/kg groups but did not differ significantly from the 0.8 g/kg group. The quantities of yellow follicles in all three diameter categories were also highest at 0.5 g/kg, increasing by 78.9%, 36.2%, and 42.4%, respectively, relative to the control. The oviduct index was numerically highest at 0.8 g/kg, whereas the quantities of 5–12 mm yellow follicles were lower than those at 0.5 g/kg.

### 3.6. Serum and Tissue Antioxidant Indices

**Figure 1:**
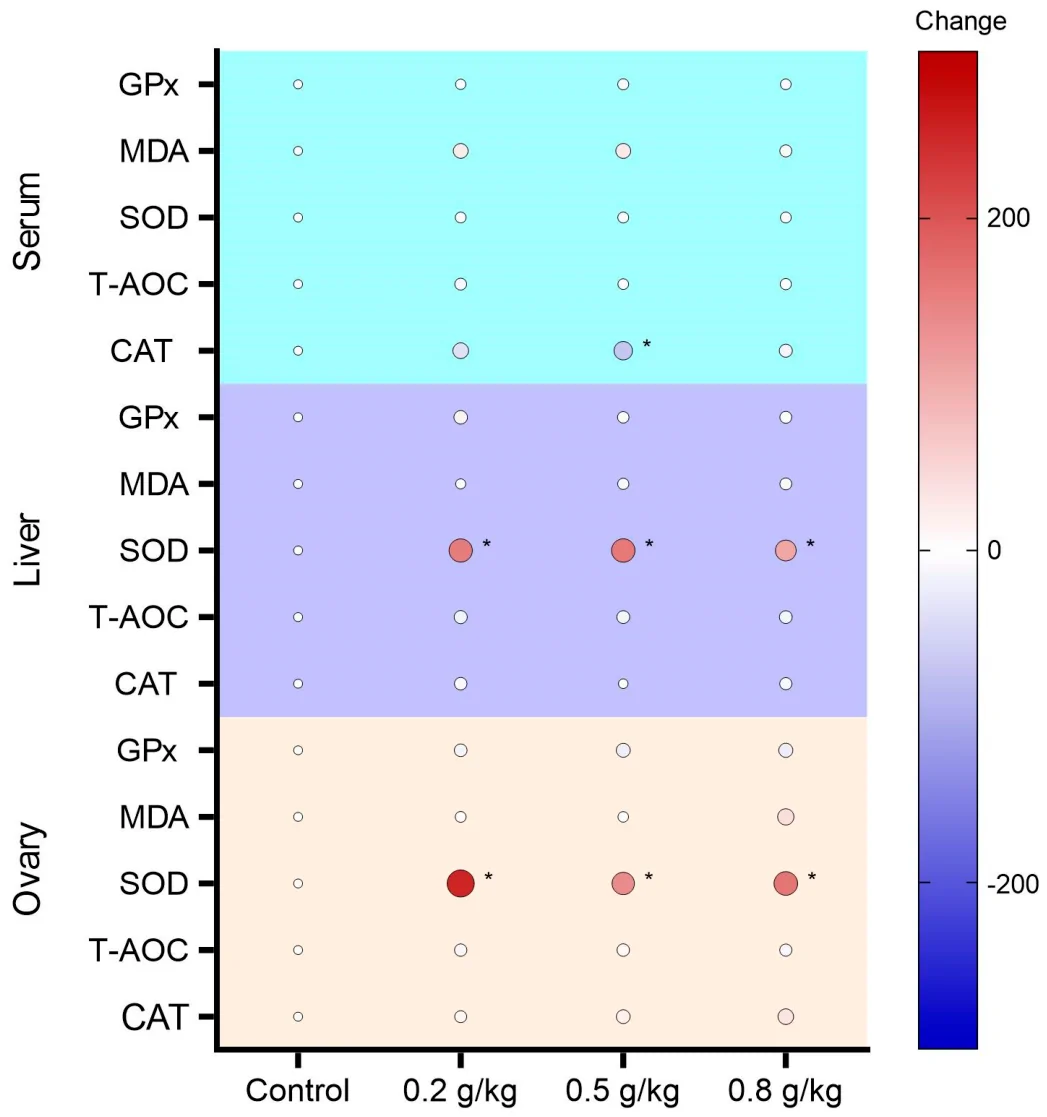
Tissue-specific antioxidant responses to dietary SYH in yellow-feathered broiler breeder hens. Note: Bubble color represents the percentage change relative to the control group, with red indicating an increase and blue indicating a decrease. Bubble size represents the absolute magnitude of the percentage change. Asterisks indicate significant differences from the control group (*p* < 0.05). Absolute numerical values with pooled SEM are provided in Table 8; the bubble heatmap is retained as a complementary visualization of tissue-specific response patterns.

Serum GPx, MDA, SOD, and T-AOC did not differ significantly among treatments; CAT activity was lowest in the 0.5 g/kg group and was significantly lower than in the control and 0.8 g/kg groups (*p* < 0.05; Table 8; Figure 1). Hepatic SOD activity increased to varying degrees in the supplemented groups, with marked increases at 0.2 and 0.5 g/kg, whereas hepatic GPx, MDA, T-AOC, and CAT did not differ significantly among treatments. Ovarian SOD activity was significantly higher in all three SYH-supplemented groups than in the control (*p* < 0.05), while ovarian GPx, MDA, T-AOC, and CAT remained unchanged. Significant antioxidant responses were therefore limited mainly to hepatic and ovarian SOD activity and serum CAT activity.

### 3.7. Follicular Gene Expression

**Figure 2:**
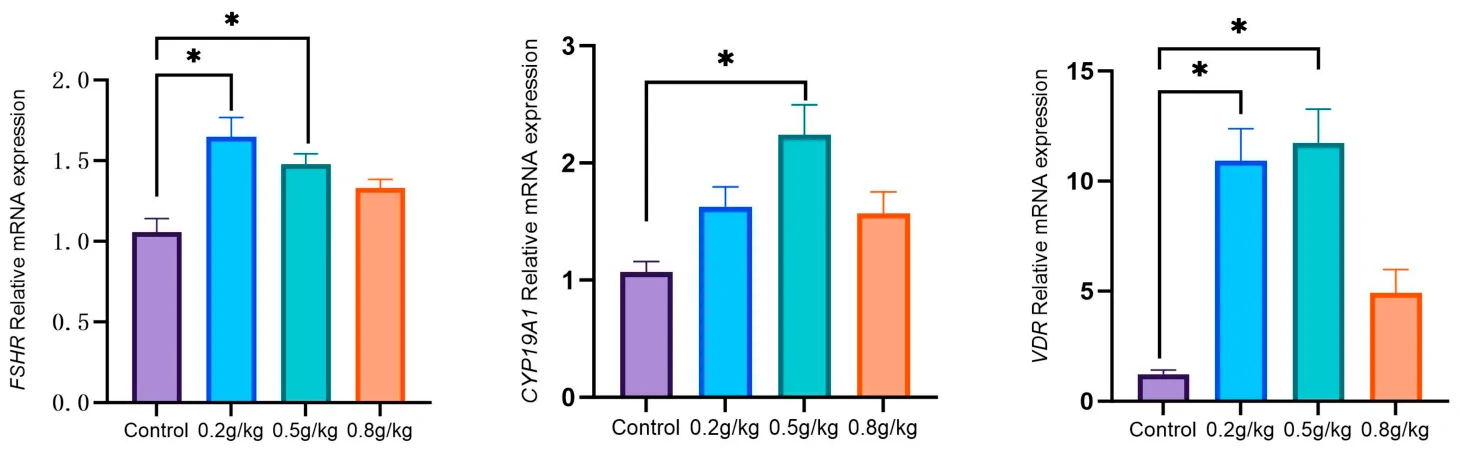
Effects of dietary SYH on the relative mRNA expression of follicular function-related genes. * indicates significant difference at *p* < 0.05 level. Note: β-actin was used as the reference gene. Data are presented as means ± standard errors. *FSHR* and *VDR* were measured in the granulosa compartment, whereas *CYP19A1* was measured in the theca compartment. All molecular analyses were performed using prehierarchical small yellow follicles (SYF; 6–8 mm in diameter).

*FSHR* mRNA expression was highest in the 0.2 g/kg group and was significantly greater than in the control and 0.8 g/kg groups (Figure 2). *CYP19A1* mRNA expression was highest in the 0.5 g/kg group and was significantly greater than in the other treatments. *VDR* mRNA expression reached 10.94 and 11.73 in the 0.2 and 0.5 g/kg groups, respectively, and was significantly higher than in the control and 0.8 g/kg groups. Although *VDR* expression at 0.8 g/kg exceeded the control value, it remained lower than at 0.2 and 0.5 g/kg. The concentration producing the highest circulating reproductive hormone concentrations differed from those producing the highest follicular gene expression.

### 3.8. Integrated Physiological Response Profile

**Figure 3:**
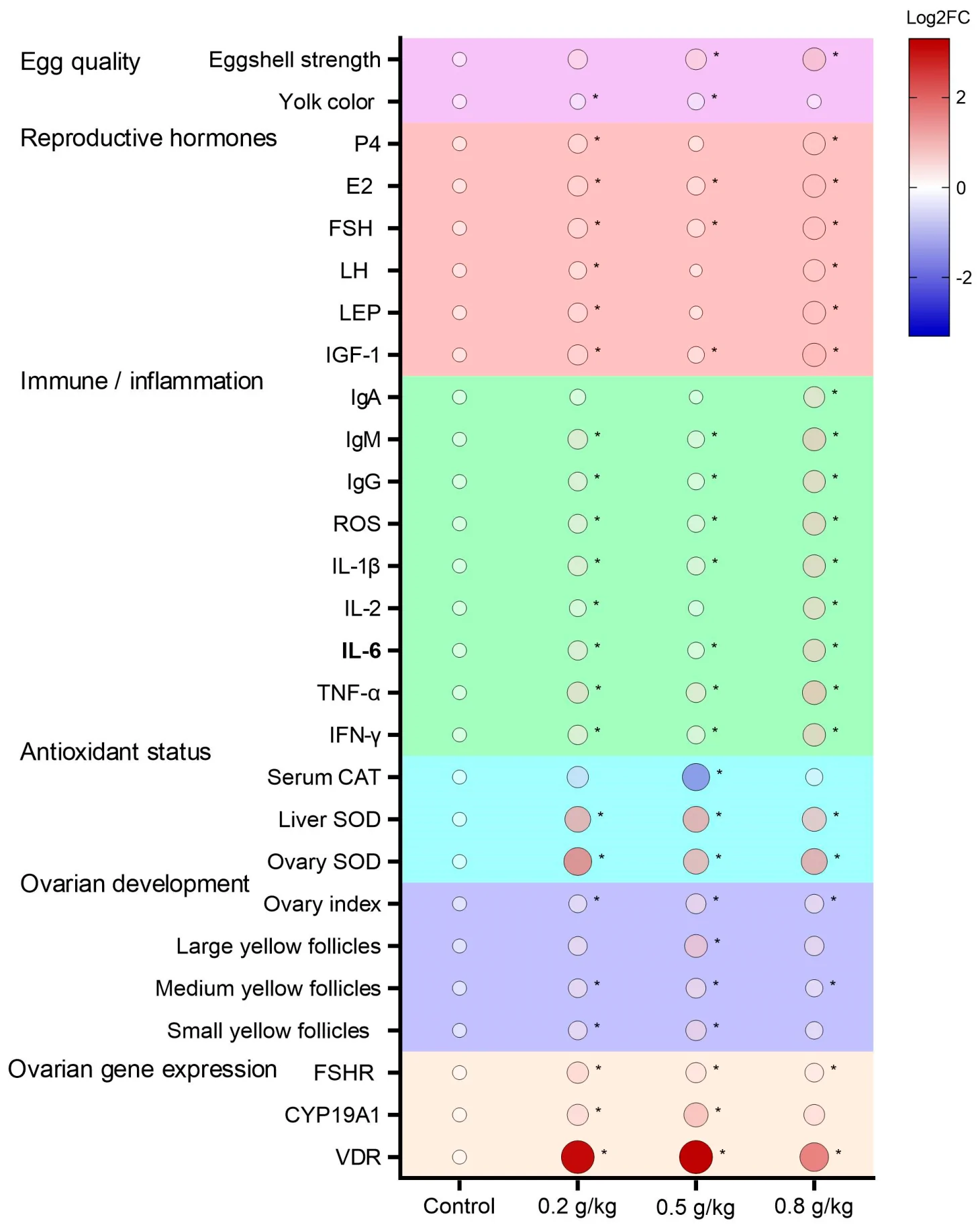
Integrated physiological response landscape to dietary SYH in yellow-feathered broiler breeder hens. Note: Bubble color represents the log_2_ fold change relative to the control group, with red indicating an increase and blue indicating a decrease. Bubble size represents the absolute magnitude of the log_2_ fold change, and asterisks indicate significant differences from the control group (*p* < 0.05). Absolute values and associated measures of variability for the individual variables are reported in Table 3, Table 4, Table 5, Table 6, Table 7 and Table 8 and Figure 2, as appropriate; Figure 3 is intended as an integrated summary of variables with different units and scales.

To facilitate comparison across endpoints, a bubble heatmap was used to integrate egg quality, reproductive hormones, immune and inflammatory indices, antioxidant status, ovarian development, and gene expression (Figure 3). Overall, the 0.5 g/kg treatment was more strongly associated with ovarian development and local follicular responses, whereas 0.8 g/kg produced stronger systemic endocrine and immune activation together with higher ROS and inflammatory mediator concentrations.

## 4. Discussion

### 4.1. Egg Quality and Hatchability

Recent studies have shown that yeast cultures, yeast fermentation products, and yeast cell wall polysaccharides can influence egg quality, antioxidant status, immune responses, intestinal microbiota, and ovarian function in laying hens [13,14,15,16,17]. Yeast fermentation products have been reported to improve Haugh unit, albumen height, and eggshell proportion in post-peak laying hens, alongside inducing changes in ovarian function and gut microbial ecology [14], whereas yeast β-glucan can improve hatching performance and both cellular and humoral immune function in breeder hens [15]. Liu et al. [6] reported that 2.0 g/kg dietary yeast culture increased eggshell strength, albumen height, and Haugh unit and improved hatchability in aged breeder layers. Zhang et al. [7] similarly showed that yeast culture supported productive performance in aged hens by improving digestive enzyme activity and intestinal health. Yalçın et al. [18] observed improved laying performance and humoral immunity after dietary yeast autolysate, whereas most conventional egg quality traits were unchanged, indicating product- and endpoint-specific responses to yeast-derived feed additives. In a meta-analysis of 18 studies in laying hens, Salami et al. [19] found that yeast cell wall-derived mannan oligosaccharides increased laying rate and egg mass and improved feed conversion, while having little effect on egg weight and only a tendency to improve eggshell thickness. Youssef et al. [20] also reported improvements in laying performance, E2, and immune responses with mannan oligosaccharides, but no significant effects on fertility, hatchability, or embryonic mortality. Collectively, these findings indicate that yeast-derived products can improve selected eggshell, production, and immune traits but do not consistently improve hatching outcomes.

In the present study, SYH increased eggshell strength without affecting albumen height or Haugh unit, which is consistent with the heterogeneous egg quality responses reported for different yeast products [18,19]. SYH also altered yolk color. The response was nonlinear, decreasing at 0.2 g/kg (low concentration) and 0.5 g/kg (intermediate concentration) and increasing again at 0.8 g/kg. Because dietary pigment content, carotenoid intake, and yolk deposition were not measured, this atypical pattern cannot be explained by the current dataset. It may reflect sampling variation, pigment deposition, or altered lipid transport and should therefore be regarded as an observation requiring further verification rather than a defined functional effect of SYH.

Hatchability did not differ significantly among treatments, consistent with reports of stable fertility and hatchability after mannan oligosaccharide supplementation [20]. Hatchability is influenced by rooster semen quality, maternal oviduct condition, hatching egg quality, and incubation conditions. Because rooster-related variables were not evaluated and the oviduct index did not differ among treatments, the present findings support an effect of SYH on eggshell quality but do not demonstrate a clear improvement in hatchability. Because all hatching eggs were evaluated in a single incubation run, variation among independent incubation batches could not be estimated. The hatchability results should therefore be interpreted as treatment comparisons under a common set of incubation conditions.

### 4.2. Ovarian Function and Serum Reproductive Hormones

FSH and LH participate in follicular recruitment, granulosa cell differentiation, ovulation, and P4 secretion. *CYP19A1* encodes aromatase and is involved in E2 synthesis, whereas *FSHR* expression determines follicular sensitivity to FSH [1,2,3]. *FSHR* has an important role in the survival of prehierarchical follicles, granulosa cell differentiation, and follicular selection in the chicken ovary [21]. *CYP19A1*-mediated aromatase activity supports the conversion of androgens to estrogens in chicken theca cells, and its transcription is regulated by factors including steroidogenic factor-1 [22]. Hernandez and Bahr [23] showed that FSH promotes P450scc expression and P4 secretion in granulosa cells from small yellow follicles, whereas epidermal growth factor attenuates this steroidogenic response. Woods and Johnson [24] further demonstrated that, within the 6–8 mm prehierarchical follicle cohort, the follicle destined for selection exhibits higher *FSHR* expression. Johnson and Lee [25] confirmed that granulosa cell responsiveness to FSH increases during follicular selection and is associated with acquisition of steroidogenic capacity, including STAR and CYP11A expression. Huang et al. [26] reported higher *FSHR* and AMHR2 expression in smaller follicles, whereas STAR, CYP11A1, and HSD3B1 increased after follicular selection, demonstrating clear developmental stage differences in local receptor and steroidogenic gene expression. Together, these studies indicate that circulating FSH alone does not fully reflect the ovarian response to FSH; follicular stage and local receptor/signaling status are also critical. In addition, LEP and IGF-1 signaling are functionally linked in the chicken ovary, and an appropriate LEP level may promote primordial follicle activation by regulating the ovarian IGF-1/IGFBP system [27]. Lei et al. [28] found that enhanced LEPR signaling reduced egg production, increased follicular apoptosis-related gene expression, and decreased ovarian IGF-I expression, indicating that LEP/LEPR effects are also dependent on signaling intensity and physiological context.

The concentration producing the highest peripheral hormone concentrations did not produce the strongest ovarian developmental response. At 0.8 g/kg, P4, E2, FSH, LH, LEP, and IGF-1 concentrations were highest, whereas the ovarian index and the quantities of 5–12 mm yellow follicles were greatest at 0.5 g/kg. Local gene responses also peaked below the highest concentration: *FSHR* expression was highest at 0.2 g/kg, whereas *CYP19A1* and *VDR* expression were highest at 0.5 g/kg. This divergence is physiologically plausible because circulating hormones represent systemic endocrine input, whereas follicular recruitment depends on developmental stage, receptor availability, granulosa cell differentiation, and local steroidogenic competence [23,24,25,26]. Thus, higher peripheral hormone concentrations do not necessarily result in a proportional increase in recruitable follicles.

Vitamin D/*VDR* signaling provides a plausible biological context for the local follicular responses observed in the present study. In laying hens, Cheng et al. reported that 1α,25-dihydroxyvitamin D3 altered *VDR* expression in granulosa and theca cells of prehierarchical follicles and modulated steroidogenesis-related genes, including *FSHR* in granulosa cells and *CYP19A1* in theca cells, together with changes in E2 and P4 production [29]. In goat ovarian granulosa cells, Yao et al. similarly demonstrated *VDR* expression and vitamin D3-dependent changes in steroidogenic activity, while *FSHR* mRNA expression decreased following vitamin D3 treatment [30]. These findings indicate that the relationship between vitamin D/*VDR* signaling and *FSHR* or steroidogenic gene expression may be context dependent. In the present study, the higher *VDR* expression at 0.2 and 0.5 g/kg broadly paralleled local *FSHR*/*CYP19A1* expression and follicular responses. However, only transcript abundance was assessed; *VDR* protein abundance or activation, *VDR*-dependent regulation of *FSHR* or *CYP19A1*, and downstream signaling were not examined. Therefore, the coordinated changes observed here support a potential involvement of *VDR*-related signaling but do not establish a causal SYH–*VDR*–*FSHR*/*CYP19A1* regulatory axis. Moreover, because SYH is a composite product containing sterols, mannan, nucleotides, and amino nitrogen, the observed ovarian responses cannot be attributed specifically to the sterol fraction. Further studies using defined sterol fractions, *VDR* inhibition or knockdown, and downstream functional assays are required to determine whether *VDR* mediates the effects of SYH on follicular development and steroidogenesis. LEP/IGF-1 signaling is also dependent on signaling intensity and physiological context [27,28]; therefore, the peak response at 0.8 g/kg should not be interpreted as uniformly favorable for follicular development.

Serum hormones were measured from a single blood sample collected between 09:00 and 11:00 at the end of the trial. The hens were not synchronized according to individual oviposition or ovulatory stage; therefore, these measurements represent a single-time-point assessment of peripheral endocrine status rather than specifically defined basal or preovulatory peak concentrations. In contrast, the ovarian index and follicle counts reflect the cumulative outcomes of follicular development over several weeks. Molecular analyses were restricted to 6–8 mm prehierarchical SYFs. This study did not quantify receptor protein abundance, downstream signaling pathways, endogenous ovarian hormone concentrations, atretic follicles, or preovulatory hierarchical follicles larger than 12 mm. Therefore, the present data demonstrate divergent concentration-response patterns between systemic endocrine parameters and local ovarian indices, but the underlying causal mechanisms remain unresolved, and a biologically optimal inclusion concentration cannot be identified.

### 4.3. Immune Responses and Redox Status

The marked increases in IgA, IgM, and IgG at 0.8 g/kg indicate an enhanced humoral immune response at the highest SYH level. Yeast culture has been shown to increase lysozyme activity and T-AOC in aged laying hens and to influence immune function through gut microbiota and yeast cell wall components [6]. Vitamin D3 supplementation can also modulate immune cells, cytokines, and reproductive hormones in laying hens and alleviate lipopolysaccharide-induced immune stress [31]. Studies of yeast cell wall components indicate that their immunological effects are not limited to reductions in pro-inflammatory mediators. Kyoung et al. [32] found that yeast cell wall improved intestinal barrier function and modulated intestinal inflammatory cytokine expression in broilers, whereas Echeverry et al. [33] showed that yeast cell wall polysaccharides regulated Toll-like receptor and Th1/Th2-related cytokine expression in lipopolysaccharide-stimulated chicken B lymphocytes. Conversely, Luo et al. [34] reported that a composite yeast culture reduced TNF-α and IFN-γ while increasing IL-4, IL-10, and TGF-β1 in late-laying hens, whereas Wang et al. [35] found that sulfated yeast β-glucan increased IFN-γ and IL-6 and promoted lymphocyte proliferation in cyclophosphamide-immunosuppressed chickens. These findings indicate that the direction of cytokine responses to yeast-derived polysaccharides depends on immune status, product composition, and dose; an increase in cytokine concentrations should therefore not be interpreted automatically as aggravated inflammation.

Qin et al. [36] reported that selenium-enriched yeast increased glutathione peroxidase and SOD activities in the liver, serum, and ovary of laying hens. In the present study, however, ROS, IL-1β, IL-2, IL-6, TNF-α, and IFN-γ increased simultaneously at 0.8 g/kg, indicating that the high-dose response was not a simple anti-inflammatory pattern. In the curcumin study of Liu et al. [4], increased immunoglobulins were accompanied by reduced IL-6, whereas the present study showed simultaneous increases in antibody responses and inflammatory and oxidative signals. In light of reports that yeast cell wall components and β-glucan can either decrease or increase specific cytokines depending on the immune model [32,33,34,35], the 0.8 g/kg treatment more likely reflects stronger systemic immune activation rather than a uniform improvement in immunity. The marked concurrent increase in ROS further suggests that this dose may increase oxidative burden.

### 4.4. Tissue-Specific Antioxidant Responses and the Appropriate Supplementation Level

Laying poultry have a high metabolic rate and can generate substantial amounts of ROS, which may induce oxidative stress and damage tissues such as the ovary and liver [37]. Oxidative stress is an important contributor to follicular atresia and ovarian functional decline in aged hens. Previous studies have shown that improving ovarian antioxidant capacity can reduce ROS and lipid peroxidation, attenuate granulosa cell apoptosis and follicular atresia, and improve reproductive hormone secretion and *CYP19A1* expression [38,39,40]. Hao et al. [41] reported that melatonin increased SOD and T-AOC, increased the quantities of medium and small follicles, increased E2 and LH, and reduced plasma ROS in aged laying hens. Using a chicken ovarian oxidative stress model, Wang et al. [42] found that oxidative stress reduced reproductive performance and follicle number and was associated with reduced SIRT1 and changes in stress-related signaling pathways including P53 and FoxO1. He et al. [43] observed that astaxanthin supplementation in 68-week-old laying hens increased ovarian NRF2 expression and antioxidant capacity, reduced follicular atresia, and increased FSH, LH, P4, and granulosa-cell *FSHR* and CYP11A1 expression. These findings further support a close relationship among ovarian redox status, follicular survival, and reproductive endocrine function, while also indicating that antioxidant enzymes and tissues need not respond in parallel.

The increases in hepatic and ovarian SOD activity indicate a selective response in superoxide removal. However, SOD converts superoxide to hydrogen peroxide, which must subsequently be removed by CAT, GPx, and related systems. Because CAT, GPx, and T-AOC did not increase consistently, MDA was not reduced, and serum CAT decreased at 0.5 g/kg, the data do not demonstrate an overall improvement in antioxidant capacity or a reduction in oxidative damage. The mechanism underlying the lower serum CAT activity was not examined. Accordingly, antioxidant modulation may be associated with the ovarian response, but a mediating role cannot be concluded from the present measurements.

Overall, SYH produced a nonlinear response. The 0.5 g/kg concentration was associated with the most favorable ovarian developmental profile among the tested treatments, whereas 0.8 g/kg produced stronger systemic endocrine, immune, and inflammatory responses without further improvement in follicle number. Because only three supplementation concentrations were tested and no formal concentration-response model was applied, 0.5 g/kg should be regarded as a candidate concentration for further validation rather than an established optimum.

## 5. Conclusions

Under the conditions of this six-week study, 0.5 g/kg of SYH was associated with greater eggshell strength than the control, the highest ovarian index values, and the greatest quantities of 5–12 mm yellow follicles among the tested groups. The 0.8 g/kg treatment produced the highest serum reproductive hormone and immunoglobulin concentrations, but ROS and cytokine concentrations were also highest and follicle quantities did not increase further. Fertility and hatchability were unaffected, and most antioxidant indices were unchanged. These findings support 0.5 g/kg as a candidate inclusion concentration for further validation rather than a confirmed optimum. The mechanism linking the peripheral endocrine peak to the local ovarian response remains unresolved.

## Figures and Tables

**Table 1:** Composition and nutrient levels of the basal diet.

Item	Content
**Ingredients (%)**	
Corn	61.55
Soybean meal	25.50
Soybean oil	1.80
Limestone	9.00
Dicalcium phosphate	1.20
DL-Methionine	0.17
Sodium chloride	0.22
Choline chloride	0.11
Sodium bicarbonate	0.17
Premix ^1^	0.28
Total	100.00
**Nutrient levels**	
Crude fat (%)	3.43
Crude protein (%)	14.78
Available phosphorus (%) ^2^	0.37
Calcium (%)	2.93
Metabolizable energy (MJ/kg) ^2^	12.92

Note: ^1^ The premix provided per kilogram of diet—vitamin A, 10,000 IU; vitamin D3, 3500 IU; vitamin E, 28 IU; vitamin K3, 2.2 mg; vitamin B1, 2.5 mg; vitamin B2, 3.5 mg; vitamin B6, 3.0 mg; vitamin B12, 0.025 mg; biotin, 0.25 mg; folic acid, 0.8 mg; niacin, 32.0 mg; calcium D-pantothenate, 12.0 mg; Fe, 90 mg; Cu, 18 mg; Mn, 90 mg; Zn, 70 mg; Se, 0.3 mg; and I, 0.5 mg. ^2^ Metabolizable energy and available phosphorus were calculated values; crude fat, crude protein, and calcium were analyzed values.

**Table 2:** Genes, primer sequences, and reference accession numbers used for RT-qPCR.

Gene	Orientation	Primer Sequence (5′–3′)	Size (bp)	GenBank
*β-actin*	F	CTTCACCACCACAGCCGAGAGAG	222	NM_205518.2
	R	CCACAGGACTCCATACCCAAGAAAG		NM_205518.2
*FSHR*	F	CTGCCAGGAGATCAAGGTGG	205	NM_205079.2
	R	TTCATGTAGTTTGGGAAGGCT		NM_205079.2
*CYP19A1*	F	CCTTCTTCACCAAAGCCCTGTC	164	NM_001001761.4
	R	GTTAGATGTGTCAAGCATAATCCGTC		NM_001001761.4
*VDR*	F	GAAAGCGATGTTCACCTGTCCG	112	NM_205098.2
	R	CTTCATCATCCCAATGTCCACG		NM_205098.2

**Table 3:** Effects of dietary SYH on egg quality in yellow-feathered broiler breeder hens.

Item	Control	0.2 g/kg of SYH	0.5 g/kg of SYH	0.8 g/kg of SYH	Pooled SEM	*p*-Value
Egg weight (g)	66.43	66.69	66.56	66.82	0.83	0.989
Egg shape index	0.7825	0.7676	0.7590	0.7612	0.0068	0.067
Eggshell strength (kgf)	2.221 ^c^	2.917 ^bc^	3.256 ^ab^	3.996 ^a^	0.287	<0.001
Eggshell thickness (mm)	0.3427	0.3366	0.3483	0.3301	0.0051	0.077
Albumen height (mm)	5.113	4.975	5.667	5.413	0.273	0.285
Yolk color	9.158 ^a^	8.513 ^bc^	8.113 ^c^	8.833 ^ab^	0.162	<0.001
Haugh unit	63.84	64.42	70.44	67.86	2.30	0.150
Yolk weight (g)	20.52	20.79	20.03	20.18	0.324	0.346

Note: Values are treatment means with pooled SEMs. Data were analyzed by one-way ANOVA followed by Duncan’s multiple range test when the overall treatment effect was significant. Within a row, means without a common lowercase superscript differ significantly (*p* < 0.05).

**Table 4:** Effects of dietary SYH on fertility and hatchability in yellow-feathered broiler breeder hens.

Item	Control	0.2 g/kg of SYH	0.5 g/kg of SYH	0.8 g/kg of SYH	Pooled SEM	*p*-Value
**Fertility (%)**	95.56	97.78	96.67	96.67	1.45	0.760
**Hatchability of eggs set (%)**	90.00	94.44	93.33	93.33	1.96	0.427
**Hatchability of fertile eggs (%)**	94.21	96.59	96.51	96.59	1.45	0.534

Note: Values are treatment means with pooled SEMs. Fertility and hatchability were calculated at the replicate-pen level (*n* = 6 pens per treatment), and individual eggs were treated as subsamples. All eggs were evaluated in a single incubation run under the same incubation conditions. Fertility and hatchability of eggs set were analyzed by one-way ANOVA. Hatchability of fertile eggs did not satisfy the homogeneity-of-variance assumption (Levene’s test, *p* = 0.030) and was therefore analyzed using Welch’s ANOVA. No significant overall treatment effects were detected; therefore, no post hoc multiple comparisons were performed.

**Table 5:** Effects of dietary SYH on serum reproductive hormone concentrations in yellow-feathered broiler breeder hens.

Item	Control	0.2 g/kg of SYH	0.5 g/kg of SYH	0.8 g/kg of SYH	Pooled SEM	*p*-Value
**P4 (nmol/L)**	9.69 ^c^	12.35 ^b^	10.30 ^c^	16.10 ^a^	0.32	<0.001
**E2 (pmol/L)**	34.77 ^d^	48.64 ^b^	42.16 ^c^	62.20 ^a^	1.50	<0.001
**FSH (mIU/mL)**	14.51 ^d^	19.60 ^b^	17.30 ^c^	25.27 ^a^	0.54	<0.001
**LH (ng/mL)**	7.71 ^c^	9.05 ^b^	7.88 ^c^	12.43 ^a^	0.27	<0.001
**LEP (ng/mL)**	2.47 ^c^	3.22 ^b^	2.53 ^c^	4.37 ^a^	0.11	<0.001
**IGF-1 (ng/mL)**	27.84 ^c^	39.02 ^b^	31.35 ^c^	52.72 ^a^	1.02	<0.001

Note: Values are treatment means with pooled SEMs. P4, E2, FSH, LH, and LEP were analyzed by one-way ANOVA followed by Duncan’s multiple range test. IGF-1 did not satisfy the homogeneity-of-variance assumption and was analyzed using Welch’s ANOVA followed by the Games–Howell multiple-comparison test. Within a row, means without a common lowercase superscript differ significantly (*p* < 0.05). FSH concentrations are reported as mIU/mL, consistent with the assay detection report; mIU denotes milli-international units (1 mIU = 10^−3^ IU).

**Table 6:** Effects of dietary SYH on serum immune- and inflammation-related indices in yellow-feathered broiler breeder hens.

Item	Control	0.2 g/kg of SYH	0.5 g/kg of SYH	0.8 g/kg of SYH	Pooled SEM	*p*-Value
**IgA (μg/mL)**	711.22 ^bc^	772.93 ^b^	692.06 ^c^	1058.95 ^a^	26.36	<0.001
**IgM (μg/mL)**	1523.46 ^d^	2053.25 ^b^	1733.00 ^c^	2783.44 ^a^	65.68	<0.001
**IgG (μg/mL)**	6.04 ^d^	7.59 ^b^	6.78 ^c^	10.10 ^a^	0.19	<0.001
**ROS (pg/mL)**	234.81 ^d^	297.38 ^b^	270.87 ^c^	416.48 ^a^	9.26	<0.001
**IL-1β (pg/mL)**	50.38 ^d^	66.76 ^b^	60.53 ^c^	85.65 ^a^	1.69	<0.001
**IL-2 (pg/mL)**	201.81 ^c^	228.54 ^b^	189.32 ^c^	326.31 ^a^	8.11	<0.001
**IL-6 (pg/mL)**	17.71 ^d^	23.14 ^b^	19.79 ^c^	30.10 ^a^	0.64	<0.001
**TNF-α (pg/mL)**	64.92 ^d^	98.96 ^b^	87.49 ^c^	129.48 ^a^	2.49	<0.001
**IFN-γ (pg/mL)**	63.55 ^d^	84.26 ^b^	76.42 ^c^	110.69 ^a^	2.45	<0.001

Note: Values are treatment means with pooled SEMs. Data were analyzed by one-way ANOVA followed by Duncan’s multiple range test. Within a row, means without a common lowercase superscript differ significantly (*p* < 0.05).

**Table 7:** Effects of dietary SYH on organ indices and ovarian follicle development in yellow-feathered broiler breeder hens.

Item	Control	0.2 g/kg of SYH	0.5 g/kg of SYH	0.8 g/kg of SYH	Pooled SEM	*p*-Value
**Liver index (%)**	2.54	2.15	1.76	1.71	0.24	0.081
**Spleen index (%)**	0.10	0.08	0.11	0.11	0.007	0.058
**Ovary index (%)**	1.85 ^c^	2.21 ^b^	2.55 ^a^	2.34 ^ab^	0.10	0.001
**Oviduct index (%)**	2.49	2.47	2.64	2.94	0.23	0.478
**Yellow follicles, 9–12 mm (n)**	3.80 ^b^	4.80 ^b^	6.80 ^a^	5.00 ^b^	0.42	0.001
**Yellow follicles, 7–9 mm (n)**	11.60 ^c^	14.60 ^ab^	15.80 ^a^	13.40 ^b^	0.60	0.001
**Yellow follicles, 5–7 mm (n)**	11.80 ^c^	14.80 ^ab^	16.80 ^a^	13.80 ^bc^	0.70	0.001

Note: Values are treatment means with pooled SEMs. Data were analyzed by one-way ANOVA followed by Duncan’s multiple range test when the overall treatment effect was significant. Within a row, means without a common lowercase superscript differ significantly (*p* < 0.05).

**Table 8:** Effects of dietary SYH on serum, hepatic, and ovarian antioxidant indices in yellow-feathered broiler breeder hens.

Item	Control	0.2 g/kg of SYH	0.5 g/kg of SYH	0.8 g/kg of SYH	Pooled SEM	*p*-Value
Serum GPx (nmol/min/mL)	483.09	490.79	465.09	471.07	14.53	0.077
Serum MDA (nmol/mL)	10.81	13.60	13.75	11.65	1.09	0.191
Serum SOD (U/mL)	123.02	119.66	119.17	126.57	17.34	0.992
Serum T-AOC (μmol Trolox/mL)	1.183	1.103	1.154	1.233	0.048	0.323
Serum CAT (μmol/min/mL)	6.99 ^a^	4.50 ^ab^	2.18 ^b^	6.09 ^a^	1.15	0.039
Serum GPx (nmol/min/g FW)	1479.75	1717.62	1554.16	1584.19	228.26	0.902
Liver MDA (nmol/g FW)	126.10	124.74	120.88	134.53	5.19	0.326
Liver SOD (U/g FW)	367.06 ^d^	940.48 ^b^	957.13 ^a^	760.59 ^c^	20.84	<0.001
Liver T-AOC (μmol Trolox/g FW)	4.400	3.840	3.880	3.893	0.195	0.170
Liver CAT (μmol/min/g FW)	3524.63	3878.10	3509.78	3238.91	225.35	0.286
Ovary GPx (nmol/min/g FW)	453.81	403.85	369.33	362.64	54.80	0.635
Ovary MDA (nmol/g FW)	123.27	119.58	120.63	173.45	26.88	0.789
Ovary SOD (U/g FW)	312.84 ^b^	1097.32 ^ab^	743.54 ^ab^	827.99 ^a^	143.20	0.003
Ovary T-AOC (μmol Trolox/g FW)	2.935	2.667	3.226	2.691	0.344	0.639
Ovary CAT (μmol/min/g FW)	67.14	72.02	79.11	89.92	7.38	0.180

Note: Values are treatment means with pooled SEMs. Variables satisfying the homogeneity-of-variance assumption were analyzed by one-way ANOVA, followed by Duncan’s multiple range test when the overall treatment effect was significant. Variables with unequal variances were analyzed using Welch’s ANOVA, followed by Games–Howell pairwise comparisons when the Welch test was significant. Within a row, means without a common lowercase superscript differ significantly at *p* < 0.05. FW, fresh weight.

## Data Availability

The data presented in this study are openly available in Mendeley Data at https://data.mendeley.com/.

## Supplementary Materials

The following supporting information can be downloaded at: https://www.mdpi.com/article/10.3390/ani16172750/s1, Table S1. Reverse transcription reaction mixture, PCR reaction mixture, and PCR cycling conditions.

## Abbreviations

ANOVA	analysis of variance
CAT	catalase
cDNA	complementary deoxyribonucleic acid
*CYP19A1*	cytochrome P450 family 19 subfamily A member 1
ddH_2_O	double-distilled water
dNTP	deoxynucleotide triphosphate
E2	estradiol
ELISA	enzyme-linked immunosorbent assay
FSH	follicle-stimulating hormone
*FSHR*	follicle-stimulating hormone receptor
GPx	glutathione peroxidase
GSH-Px	glutathione peroxidase
IFN-γ	interferon-γ
IGF-1	insulin-like growth factor-1
IgA	immunoglobulin A
IgG	immunoglobulin G
IgM	immunoglobulin M
IL-1β	interleukin-1β
IL-2	interleukin-2
IL-6	interleukin-6
LEP	leptin
LH	luteinizing hormone
LYF	large yellow follicle
MDA	malondialdehyde
M-MuLV	Moloney murine leukemia virus
P4	progesterone
PBS	phosphate-buffered saline
PCR	polymerase chain reaction
RNA	ribonucleic acid
ROS	reactive oxygen species
ROX	6-carboxy-X-rhodamine
SEM	standard error of the mean
SOD	superoxide dismutase
SYF	small yellow follicle
SYH	sterol-enriched yeast hydrolysate
T-AOC	total antioxidant capacity
TNF-α	tumor necrosis factor-α
*VDR*	vitamin D receptor
FW	fresh weight
