# Supplementary material for: Effects of Dietary Sterol-Enriched Yeast Hydrolysate on Ovarian Development, Reproductive Endocrine Status, Immune-Inflammatory Responses, Antioxidant Indices, and Egg Quality in Late-Laying Yellow-Feathered Broiler Breeder Hens
Source: Animals (Basel). 2026 Sep 2;16(17):2750. doi: 10.3390/ani16172750 (PMC13565570; doi:10.3390/ani16172750)
Supplement: Supplementary file 1 [file animals-16-02750-s001.zip › animals-4510809-supplementary.pdf]

Table S1 Reverse transcription reaction mixture, PCR reaction mixture, and PCR cycling conditions

**Reverse transcription system**	**Volume in a 20-μL reaction**
5× Reaction Buffer	4 μL
Primer	1 μL
RNA	5 μg
RiboLock ribonuclease inhibitor	1 μL
RevertAid Moloney murine leukemia virus (M-MuLV) reverse transcriptase	1 μL
10 mM deoxynucleotide triphosphate (dNTP) mix	2 μL
Ribonuclease/deoxyribonuclease-free double-distilled water (ddH_2O)	To 20 μL
**PCR reaction system**	**Volume in a 20-μL reaction**
Complementary deoxyribonucleic acid (cDNA)	100 ng (1 μL)
Forward primer	0.5 μL
Reverse primer	0.5 μL
SYBR Green mix	10 μL
6-carboxy-X-rhodamine (ROX) reference dye (50×)	0.4 μL
Ribonuclease/deoxyribonuclease-free double-distilled water (ddH_2O)	To 20 μL
**Reaction temperature**	**Time**
94 °C	30 s
94 °C	5 s
60 °C	30 s
95 °C	15 s
60 °C	1 min
Increase by 0.3 °C every 15 s	
95 °C	15 s
